# Review of Importance of Weather and Environmental Variables in Agent-Based Arbovirus Models

**DOI:** 10.3390/ijerph192315578

**Published:** 2022-11-24

**Authors:** Luba Pascoe, Thomas Clemen, Karen Bradshaw, Devotha Nyambo

**Affiliations:** 1 Nelson Mandela African Institution of Science and Technology, Arusha P.O Box 447, Tanzania; 2 Department of Computer Science, Hamburg University of Applied Sciences, Berliner Tor 7, 20099 Hamburg, Germany; 3 Department of Computer Science, Rhodes University, Grahamstown 6139, South Africa

**Keywords:** dengue fever, arbovirus, climatic variables, environmental variables, agent-based modeling, temperature, precipitation, humidity

## Abstract

The study sought to review the works of literature on agent-based modeling and the influence of climatic and environmental factors on disease outbreak, transmission, and surveillance. Thus, drawing the influence of environmental variables such as vegetation index, households, mosquito habitats, breeding sites, and climatic variables including precipitation or rainfall, temperature, wind speed, and relative humidity on dengue disease modeling using the agent-based model in an African context and globally was the aim of the study. A search strategy was developed and used to search for relevant articles from four databases, namely, PubMed, Scopus, Research4Life, and Google Scholar. Inclusion criteria were developed, and 20 articles met the criteria and have been included in the review. From the reviewed works of literature, the study observed that climatic and environmental factors may influence the arbovirus disease outbreak, transmission, and surveillance. Thus, there is a call for further research on the area. To benefit from arbovirus modeling, it is crucial to consider the influence of climatic and environmental factors, especially in Africa, where there are limited studies exploring this phenomenon.

## 1. Introduction

Infectious diseases such as dengue, malaria, chikungunya, Zika, and yellow fever, to mention a few, are emerging as a worldwide challenge in public health. They occur rapidly and spread to large areas in a relatively short space of time, thus leading to an increase in mortality rates globally as well as in Sub-Saharan Africa (SSA) [1,2,3,4,5,6,7].

In addition, globally, mosquito-related diseases which include dengue fever, malaria, chikungunya, Zika, yellow fever, and others have turned out to be a major public health concern, with estimates that half the approximated world population of 9 billion is in danger of contracting an arbovirus infection by 2050 [7,8,9].

**Figure 1:**
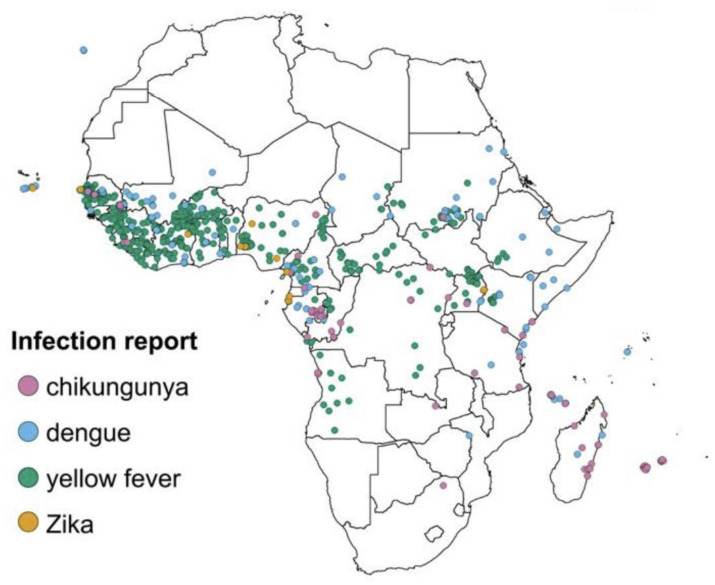
Locations of reported chikungunya, dengue, yellow fever and Zika infections in Africa. Source [12].

Arthropods are abundant in tropical and subtropical regions, resulting in the high proportion of arboviruses in these regions [6]. The global distribution of viruses has caused some arboviruses to be endemic in specific regions of the world. Global warming, deforestation, and urbanization have resulted in a dramatic increase in vector-borne diseases in the world due to the rapid expansion of vector habitats [10]. The transportation of infected mosquitos and their eggs to various new ecological niches is increased through international travel, while shipping and industrialization can also facilitate virus-vector–human host interactions, causing outbreaks owing to lower herd immunity. Herd immunity in a community is acquired when a high percentage of the community is immune to disease through vaccination or previous infection. Lack of enough approved dengue vaccination strategies, different dengue strains, and lack of efficient and sustainable vector control strategies contribute to lower herd immunity. Furthermore, the active circulation of multi-serotypes of dengue make it hyperendemic in many countries. It is posited that, decrease in cross-immunity is among the contributory factor to large dengue outbreaks [11]. Outbreaks happening in new areas for the first time mostly tend to involve immunologically naïve populations resulting into high rates of attack [11]. Marchi, Trombetta, and Montomoli [10] report that “the greatest health risk of arbovirus emergence comes from extensive tropical urbanization and colonization of this expanding habitat by the highly anthropophilic mosquito, *Aedes aegypti*, together with the recent invasion into the Americas, Europe and Africa of *Aedes albopictus* that could enhance transmission of these viruses in temperate regions”. Figure 1 shows the distribution of dengue, chikungunya, yellow fever and Zika infections in Africa [12].

Dengue virus (DENV) has spread throughout the world and is regarded as the most menacing arbovirus disease [6,7,13,14]. A study by Marchi, Trombetta, and Montomoli [10] reports on the existence of high levels of dengue endemic transmission in Americas, the western Pacific, and south-east Asia with around 4 billion people at risk of being infected. Additionally, DENV has been identified in Europe since 2010 and in 2012 an outbreak was recorded in Madeira. It has been stated that DENV’s four serotypes are spreading across Africa, although the most frequently reported serotype is DENV-2 [15]. A study by Mordecai et al. [16] reports the possible shift in the disease burden from malaria cases to arboviruses such as dengue and chikungunya in SSA countries due to climate change and urbanization. Climate change provides suitable environment that favors *Ae. Aegypti* while providing unfavorable environments for *Anopheles gambiae* [16,17]. The shift into vector-borne diseases burden is already witnessed in Sudan in which chikungunya, Rift Valley fever and dengue cases have been reported in Red Sea state, Kassala state and Darfur region [17]. It is alarming when this shift in disease burden is happening in Africa because most of the communities are poor, the governments and health systems do not have control program, health policies or local capacity for early detection and response to arboviral diseases as soon as they emerge [17]. In Tanzania, DENV has also been reported since 2010 [18] with a substantial rise in dengue fever cases in April and May of 2019 [19]. Dar es Salaam was declared an epicenter of the dengue outbreak that occurred in 2019 and the preceding outbreaks even though there have been occurrences in different regions [19].

Rweyemamu et al. [20] and Mahmood et al. [14] outline that, it is crucial to establish good monitoring, investigation, and reporting systems for infectious disease incidences which will play a major role in the management of existing diseases as well as contribute to an adaptive and flexible response to new and emerging diseases. Every year, millions of individuals are at risk of serious illness as a result of new infections. A lack of infrastructure for timely collecting, reporting, and analyzing epidemic data has posed a significant threat to public health security at the local, regional, and national levels [14,20]. In addition, it has been noted that policymakers, epidemiologists, and other related stakeholders depend highly on accurate and timely information to make informed decisions that will improve the well-being of the nation as well as the national healthcare system [14,21,22]. Furthermore, real-time data provide decision-makers with the knowledge required to respond effectively to the population’s health necessities; as a result, the value of timely and accurate data can be observed [23].

### 1.1. Background Concepts

#### 1.1.1. Dengue Fever

Dengue fever is a serious disease that affects tropical and subtropical countries worldwide [24,25]. The disease is a major public health concern with economic implications. Mahmood et al. [14] reported that 3.98 billion people in 128 countries are at risk of contracting dengue [13,26,27]. An annual estimate of over 105 million dengue cases is reported [11,28]. A study by Cattarino et al. [28] further estimates that most dengue disease burden was concentrated in South and Southeast Asia valued to about 58%, 26% occurring in SSA mostly concentrated in Central and Eastern Africa, and Latin America had an estimation of 16% of the global burden. Although the information about the prevalence of viral diseases is limited, it is noted that several studies have been carried out to determine the prevalence and spread of dengue infections and outbreaks in Tanzania [29]. Conversely, the literal roles of climatic, socio-environment and ecological variables in the spread of dengue have not been extensively investigated [30].

The health system in SSA lacks the capacity for adequate disease reporting and timely response due to unreliable data [22,31]. The untimeliness, incompleteness, inconsistency, and inaccuracy of the data, among other things, contribute to data unreliability [22,31].

*Ae. Aegypti* is the main vector for dengue, chikungunya, yellow fever and Zika [32,33]. *Aedes albopictus* which is a secondary vector for dengue, chikungunya, yellow fever and Zika is also present in some regions in Africa such as Cameroon, Gabon, Nigeria, Congo, Côte d’Ivoire, Central African Republic, Sudan and South Africa [16,34,35]. *Ae. Aegypti* is considered a domestic vector because it is dominant in urban areas, while *Ae. Albopictus* is mostly found in rural, peri-urban settings and forest areas in tropical, subtropical and temperate regions of the world [10,36,37,38]. The difference in distribution of these species is related to their behavior based on host preference, blood feeding, preference for vegetation, suitable conditions for resting and ovipositioning [35,36,37]. *Ae. Albopictus* can be quite competitively dominating when it coexists with *Ae. Aegypti* [16].

Some studies provide information on dengue transmission and risk factors in African countries such as Tanzania [29,34,39,40], Kenya [16,34], Uganda, Mozambique, [16] Sudan [17,35,41], Côte d’Ivoire, Cameroon, Gabon [16,34,42] Senegal, Nigeria, and Sierra Leone [16,34]. There is a growing evidence that in SSA epidemic and endemic transmissions of *Aedes*-transmitted arboviruses such as dengue, chikungunya and others have regularly occurred but have been undiagnosed or misdiagnosed as malaria [16]. The increase in climate suitability for *Aedes*-transmitted arboviruses pose an under-recognized public health burden to the African countries [16]. Among the factors that contribute to dengue infection transmission are climate change, urbanization, globalization of trade and travel, increased population density, and the unavailability of effective prevention control methods [16,43]. Furthermore, Dumont et al. [44] identified risk factors for dengue fever in which individual factors such as age, sex, and level of education were important in determining the risk of contracting DENV. Dumont et al. [44] indicated that household factors such as the number of people residing in a room as well as the size of the community increased the chance of contracting dengue fever. Socioeconomic and demographic factors included low income while living and traveling to endemic areas increased the chances to contact DENV. The existence of anthropogenic breeding areas for *Ae. Aegypti* (such as disposed of plastic containers and car tires that are not used), presence of vegetation density (such as leaf axils, fallen leaves, flower brats, and tree holes), height above sea level and existence of animals that are associated with dengue and chikungunya viruses’ transmission made up the environmental factors [7,44]. Thus, humidity, temperature, human migration, wind speed and sanitation contribute to the epidemic conditions in areas affected by dengue and chikungunya [6,44,45,46].

The World Health Organization (WHO) recommends integrated vector control strategies to prevent and control dengue infections that can lead to outbreaks [13,23,43]. These control strategies can be through environmental management control by eliminating the potential breeding sites for mosquitoes, namely stagnant water sites as well as chemical and biological controls. According to the literature, most of the control and prevention strategies are based on mosquito vector density management [23,43], which is influenced by different factors among them being climatic and environmental factors.

#### 1.1.2. Agent-Based Modeling

Agent-based modeling (ABM) presents a useful approach for processing health data and producing simulations that provide meaningful information for decision-makers concerning different disease outbreaks, reporting, and containment. ABM describes the underlying social/epidemiological system as well as provides a versatile and powerful platform for modeling different healthcare interventions and answering a wide range of policy-making questions [5]. As a result, ABM is regarded as a relatively new simulation technique that is gaining popularity and supports many varied applications across different fields.

ABM exhibits several benefits that make it suitable for modeling real-world systems, including bounded rationality, emergent behavior, and the bottom-up approach in modeling which results in a macro-system based on sub-system interactions. Other benefits include the heterogeneity and discrete nature of agent-based models which make them suitable for modeling heterogeneous populations; networked interactions among agents, as well as the completeness of the agent-based models since they are well detailed as they provide both individual and aggregate level detail simultaneously. Furthermore, agent-based models are very flexible and allow the incorporation of randomness into the models with agents’ decisions being based on probability rather than being strictly deterministic [47,48,49,50].

ABM follows a bottom-up approach which makes it a flexible and powerful tool for modeling complicated systems that have many interacting components [51,52]. In epidemiology, due to the increased complexity of the systems that need to be analyzed and modeled based on their interdependencies, ABMs complement statistical and mathematical models making them more accurate and suitable for predictions [53,54,55]. Complex systems and complexity lead to the rise of unpredictable patterns or global structures as a direct outcome of local-level procedures [52]. Furthermore, the organization of data into databases at finer levels of granularity and the advancement in computation power makes ABM more favorable than system dynamics, spatial interaction, and diffusion models which cannot handle the heterogeneity of data although they have successfully predicted macro-level behavioral patterns. Furthermore, ABM is capable of simulating individuals, their interactions, and the resulting consequences [47,49,53].

Several studies have modeled different aspects of dengue epidemics. Jacintho et al. [56] observed the behavioral spread of dengue fever based on how the simulation agents interact with their environment. Jindal and Rao modeled mosquito-borne diseases including malaria, dengue, and chikungunya by considering the human mobility patterns as a major source of the spatial movement of infections [5]. Mahmood et al. [14] modeled the dynamics of the population together with how both humans and mosquitoes interact intending to assist epidemiologists to explore and predict infectious disease transmission and spread. In this study, the benefit of using ABM over the compartmental model has been explored and SEIR model is utilized through a proprietary AnyLogic framework. A network type based on distance is used in which messages are passed to accomplish the interaction of host and vector agents. The whole population of the host is initialized in a susceptible state rather than distributing the population with different initial states which can enhance realistic model configurations. Pathogen’s structure and behavior is restricted to serotype initialization that is DENV1, DENV2, DENV3 or DENV4 and the key parameters such as survivability, infectivity, incubation period plus transmissibility. The framework has not considered the cross immunity of the serotypes. The framework does not support a large population of a million or more agents and lacks a mobility layer that can incorporate the movements of both host and vector layers.

A study by Stiner and Chellamuthu [57] used ABM to model the complex agent mosquito with its spatial-temporal attributes; the life cycle of a mosquito is modeled to show the effect of temperature on the different stages of mosquito development depending on the different types of mosquitoes. Then, different control strategies are advised for the different diseases caused by the mosquitoes.

A study by Mniszewski et al. [58] used agent-based modeling to simulate the spread of infectious diseases through the population using EpiSims software. EpiSims uses three sets of information (population, location and movement of individuals between locations) to simulate a disease spread in a geographical area. The study leveraged the usefulness of various models by suggesting a hybrid network patch model to provide insights into the effect of variable probabilities in the infection model on agent-based modeling. However, this study does not capture the host–vector interactions [5].

Other studies have modeled how temperature, rainfall, and humidity affect the spread of arboviruses [59]. An increase in temperature favors the virus’ replication and a shorter extrinsic incubation period, which results in increased density of infected vectors [59,60]. Extreme heat threatens adult mosquito survival rates and more quickly dries out breeding grounds, reducing mosquito numbers. Increased breeding sites and decreased mosquito mortality are also observed with an increase in precipitation and humidity levels [60], while higher precipitation may minimize mosquito numbers by washing out the immature stages [61].

It is thus important to include climatic and environmental factors in disease modeling because these factors have a great influence on infectious disease outbreaks, spread, and surveillance. This paper presents a review of the involvement of climatic and environmental factors in modeling arbovirus diseases globally and in the African context.

## 2. Materials and Methods

A search strategy was developed and used to search for articles in various databases resulting in 2334 published articles on infectious disease modeling being collected. The studies included 1875 journal articles, 131 published books, 89 book chapters, 86 conference articles, 93 dissertations, 15 conference abstracts, 9 book reviews, 8 systematic reviews, 11 government publications, 10 theses, 3 guidelines, and 4 case reports. All these articles were obtained from searches conducted in four databases, namely, Rsearch4Life, PubMed, Scopus, and Google Scholar.

### 2.1. PubMed Search

A PubMed database search conducted on 1 April 2022 yielded 108 results which fulfilled the designated search query. The search query was created using MESH by the criteria that are presented in Appendix A.

### 2.2. SCOPUS Search

A SCOPUS database search conducted on 2 April 2022 yielded 1023 results which fulfilled the designated search query as given in Appendix B.

### 2.3. Research4Life Search

A search using the Research4Life database was conducted on 2 April 2022 and yielded 230 results which were sorted by relevance. The selected language was English, and the results included items from outside the Research4Life library as well to be able to capture other literature that satisfies the search query. The following search query was used.

(Dengue OR “Dengue Fever” OR “Viral Disease*” OR “Viral Infection*” OR “Hemorrhagic Fever” OR “Aedes Aegypti” OR “Aedes Albopictus” OR “Dengue Virus” OR DENV) AND Climat* OR Temperature OR Rainfall OR Humidity OR “Relative Humidity” OR Weather) OR (Environment* OR Vegetation OR Forest* OR “Forested Area*” OR Woodland* OR Forestland* OR Wetland OR Swamp* OR “Environmental Factor*”)) AND (“Agent-Based Model*” OR “Agent-Based Model*” OR “Modeling Agent-Based” OR “Multi-Agent Systems” OR “Individual-Based Systems”).

### 2.4. Google Scholar Search

A search via Google Scholar was conducted on 2 April 2022 and yielded 1050 results using the anytime field option and sorting by relevance. Of the 1050 results, only 973 were retained for further analysis. The following search query was used.

(Agent-based Model OR Multi-Agent-Based Model OR Individual-Based Model) AND (Dengue* OR DENV OR Aedes*) AND (environment* OR climat* OR weather OR spatiotemporal OR rainfall OR temperature OR precipitation OR humidity) AND (Africa OR Sub-Saharan Africa).

All the search results were exported to RAYYAN for screening and duplicate detection, during which 902 exact duplicates were identified and one article had four exact duplicates. There were 409 duplicates that were automatically resolved, while 493 duplicates had to be manually resolved as the RAYYAN software did not identify 100% similarity in the articles. After duplicate removal, title and abstract screening were performed and 136 articles qualified for full-text screening.

### 2.5. Inclusion Criteria

Only studies that met the following criteria were included:

i. Those that investigated the effects of climatic factors or environmental factors (for example rainfall, temperature, humidity, landscape type, mosquito habitats) on the incidence, transmission, and modeling of infectious diseases.
ii. Those related to arboviruses, especially in Africa.
iii. Those that involved modeling of arbovirus disease.
iv. Studies that were published in any year.
v. Articles that were published in English.

## 3. Results

**Figure 2:**
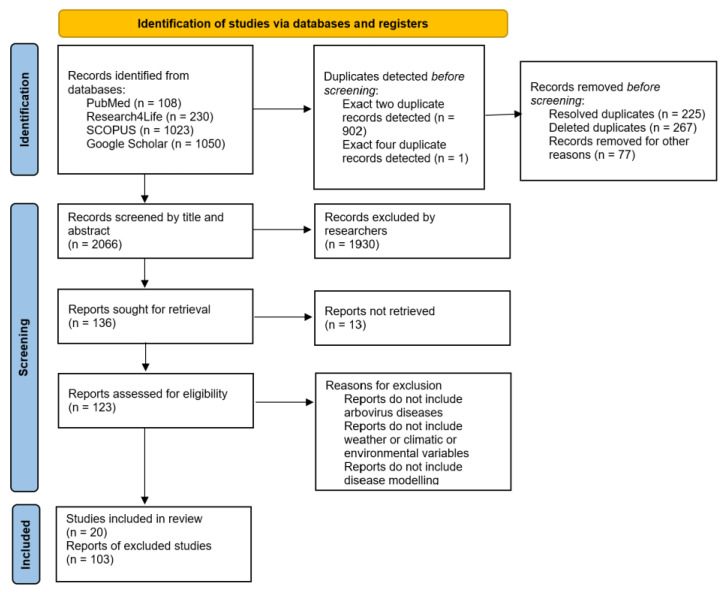
The PRISMA flow diagram.

As shown in Figure 2, which represents the PRISMA flow diagram, 20 articles have been included in this review. These articles contained information on either climatic variables and environmental variables with their relation to arbovirus diseases in Africa or globally or infectious disease modeling using ABM or other techniques. The articles are summarized in Table A1 in Appendix C. This review aimed to determine the influence of environmental variables such as vegetation index, households, mosquito habitats, and breeding sites, and climatic variables including precipitation or rainfall, temperature, and relative humidity on dengue disease modeling using an agent-based model in an African context and globally. Due to the limited availability of literature, the scope of the review had to be expanded to include all literature obtained from the search which met the stated inclusion criteria. Most studies on dengue modeling and ABM have been conducted in other countries rather than African countries. From Table A1, six studies were conducted in an African context, while twelve studies were from other continents, one study covered a global context, and one study was carried out in a virtual environment. Six studies have either modeled malaria using ABM or have incorporated climatic or environmental factors when modeling malaria [59,62,63,64,65,66,67,68]. Studies included in the review have satisfied the stated inclusion criteria and have either used ABM or included the component of climatic or environmental variables and their influence on infectious disease transmission. Three studies modeled *Ae. Aegypti* or *Ae. Albopictus* mosquitoes which apart from being dengue vectors, are responsible for spreading other pathogens such as Zika, yellow fever, chikungunya, and others [24,32,62,69,70,71,72]. Ten studies modeled dengue disease using either ABM or other modeling techniques as represented in Table A1 in Appendix C [14,25,26,73,74,75,76,77,78]. Eighteen studies have considered climatic factors in modeling the diseases, thirteen studies have involved environmental factors in modeling the diseases, out of which, eleven studies have included both climatic and environmental factors. Furthermore, eleven studies have modeled diseases using ABM, four studies used machine learning, one study used time series, and four studies used mathematical modeling.

### 3.1. Temperature

Various temperature metrics have been used in different studies to establish their relationship with arbovirus incidences [59,60]. Metrics such as mean temperature, minimum temperature, maximum temperature, air temperature, and water temperature have been identified to influence mosquito development, especially during their aquatic stages that is egg, larvae, and pupae stages [14,24,25,32,68,75,76,78,79]. The range of *Ae. Aegypti* is tremendously constrained by yearly minimum low temperatures below which its eggs are inviable [69], while on the other hand, *Ae. Aegypti* abundance could increase or decrease depending on the rate of warming as well as the magnitude of temperature increase [6,7,69]. The optimal temperature range suitable for adult Aedes mosquitoes is established in different studies to be between 15 °C and 30 °C which is within the range of 10 °C to 35 °C. Deza-Cruz [70] when citing Goindin et al. [80] reported a study in the Caribbean islands modeling dengue disease and chikungunya disease using machine learning algorithms in which it was found that half the *Ae. Aegypti* female mosquitoes in an experiment survived for more than 38 days when the temperature was 27 °C, while the other half lived for fewer than 25 days when the temperature was 24 °C or 30 °C [70,80]. The number of days a female *Ae. Aegypti* can transmit the dengue disease is strongly influenced by the temperature; at the optimal temperature of 27 °C infectious days can be as high as 21 days, while this decreases when the temperature is 20 °C and is around 20 days when the temperature is 30 °C [70,80].

The study by Talaga et al. [24], when modeling *Ae. Aegypti* using an inference approach, showed that urban areas which were dominated by warmer and dry surroundings leading to less vegetation cover were associated with lower productivity rates, survival rates, and dispersal compared to the rural surroundings but due to the effect of warmer temperature on reducing the immature development time, more offspring could be produced resulting in the production of more adult mosquitoes per year [24]. In slightly urbanized sites, the relative importance of temperature was high, and it positively influenced the abundance of *Ae. Aegypti* immatures [24].

Higher temperatures speed up the growth of larvae into adult vectors, accelerate the rate at which they bite, and shorten the time needed for extrinsic incubation. As a result, vectors become infectious earlier and bite more frequently resulting into an increase transmission of diseases [73,81]. However, to counteract the beneficial effect of vector abundance, higher temperatures may shorten the vector survival time. At 20 °C the extrinsic period is estimated to be 29.6 days, while at 30 °C, the extrinsic period is 5.2 days [70,82].

On the other hand, a study by Kapwata et al. [64] which explored rural hospital admissions for diarrheal disease, pneumonia, malaria, and asthma with climatic factors using wavelet transform analysis found a positive correlation between high temperatures with an increase in malaria transmission as mosquitoes develop faster as temperature increases and they can feed at shorter intervals because blood meals are more rapidly digested thus increasing the risk of malaria transmission [64].

Additionally, a study in South Sudan by Mukhtar et al. [66] when modeling malaria assessed the impact of temperature on the dynamics of the mosquito population using a compartmental mathematical model. Mukhtar et al. [66] reported that temperatures of 10 °C–35 °C are most favorable for mosquitoes breeding and breeding rates decrease with temperatures outside of this range. The study points out that because mosquitoes are heterothermic organisms and therefore unable to regulate their body temperatures on their own, their body temperature is mostly influenced by the environment in which they are found [66]. This study found a correlation between mosquitoes’ abundance and a mean monthly temperature range of 25 °C–30 °C, when temperatures were consistently above 10 °C mosquitoes were active but they were sedentary when temperatures reached 35 °C. A daily temperature range of 20 °C–35 °C was found to be ideal for the progression of mosquitoes and the spread of malaria and thus concluding that temperature ranges of 25 °C–30 °C were more suitable for mosquito progression at all stages in their life cycle. Immature mosquitoes were more sensitive to temperatures at 25 °C than mature mosquitoes [66].

Mulyani et al. [76] developed an ABM using NetLogo to simulate the spreading of dengue fever by observing related parameters and interactions, agents’ behaviors and interactions, and environmental factors. The model was calibrated with meteorological data from the Dramaga region in Bogor in 2015. Mosquitoes were inactive for a temperature range between 0 °C and 10 °C and temperatures above 39 °C. The random flying behavior of mosquitoes was observed at temperature ranges greater than 10 °C and less than 20 °C. Between 20 °C and 39 °C mosquitoes could fly randomly and exhibit bite behavior, while reproduction was possible at temperatures between 25 °C and 27 °C. Mosquitoes die at temperatures less than 0 °C and greater than 41 °C. The model showed the sensitivity of temperature on the behavior of modeled agents and their interactions with the environment. The human infection in the model showed a 16% decrease in infection in the first period January–June compared with the second period July–December 2015, which correlated with the human infection at Dramaga sub-district which decreased by 56% in the two categorized 6-month periods [76].

A study by Mahmood et al. [14] modeled dengue infection in Pakistan using an ABM and observed a similar trend in dengue cases prevalence between the actual values and the simulated results confirming dependency of dengue transmission on temperature and increased biting rate of *Ae. Aegypti* with temperature increase thus giving rise to dengue cases [14].

A study by Rodríguez [78], developed an ABM, which integrated a geographic information system to simulate the spread of dengue fever disease in the Central Valley of Costa Rica. Individual interactions in a geospatial context were analyzed with different variables such as precipitation, temperature, socio-economic and demographic variables to identify the factors that affected the rates of dengue fever in the targeted urban environments of Costa Rica. The temperature ranges in which mosquitoes fed and bred was 20 °C–39 °C with an optimal survival temperature of approximately 27 °C–31 °C. *Ae. Aegypti* was inactive below 10 °C and above 39 °C. Meanwhile, temperatures below 0 °C and above 41 °C would result in the death of the mosquito. In this study, an increase in dengue cases during winter seasonal months of May to November was observed, while the number of cases decreased in the summer months of December to April. Therefore, high temperatures and high precipitation patterns greatly affected the highest proportion of reported dengue fever cases in the region [78].

### 3.2. Precipitation

The reviewed studies indicated a higher transmission of arboviral diseases during the rainy periods with higher temperatures and lower diseases transmission during the dry season [24,67]. The abundance of *Ae. Aegypti* immatures in moderate and highly urbanized sites was found to be positively influenced by the size of the aquatic habitat and the amount of precipitation. Increased rainfall was determined as a risk factor for arbovirus outbreak during the study period by Rumisha et al. [67] when modeling malaria using Bayesian regression models and machine learning models. Low-lying regions and wetlands created suitable habitats for mosquitoes and increased the risk of disease transmission [69,72].

Flooding can be disadvantageous to vector populations, causing a reduction in the mosquito population by destroying breeding sites and aquatic stages of mosquitoes [66]. Consequently, an inverse relationship can exist between precipitation and mosquito populations where breeding sites have been washed away with flooding water [70].

Lack of rainfall and drought are also associated with lower disease incidence, because drought usually results in a reduction in mosquito populations [67]. A study in Rufiji recorded low transmission of malaria during the dry season of the year and higher transmission during rainy periods with higher temperatures [67].

*Ae. Aegypti* breed in containers with clean water which are mostly located in human residences. Thus, changes in precipitation could affect the availability of these vector breeding sites depending on their location either indoors or outdoors rain filled objects and as a result influence the vector abundance [73]. A study by Anders [73] modeling dengue disease using a mathematical model in south Vietnam reported that prominent seasonal peaks which coincided with the rainy season were exhibited by dengue disease and every year it was found to lead to tens of thousands of hospitalizations [73,83,84].

A study by Deza-Cruz [70] citing Alto and Juliano [85] reported that higher temperatures with no drying conditions increased the number of adults produced whereas higher temperatures with drying conditions decreased the number of adults produced. *Aedes* mosquito abundance increased with rain but decreased with more rain as moderate rain could help in the creation of breeding sites but prolonged or very heavy rain might have destroyed the sites and wiped away the immatures or kill mosquitoes [70,81,85]. An increase in *Aedes* mosquito abundance positively associates with increased dengue and chikungunya transmissions, while its decrease is associated with low dengue and chikungunya incidences [70].

A study in Mali and Burkina Faso that modeled malaria using an impact model found that a one-month rainfall threshold of 80 mm followed by two monthly rainfall thresholds of at least 60 mm would result in a malaria epidemic [65]. Low or excessive levels of rainfall negatively affected the survival probability of immature mosquitoes [66] because the mosquito birth rate decreased towards zero when rainfall was minimal and increased with an increase in rainfall but excessive rainfall might lead to washout of the immatures and thus affect the mosquito birth rate. However, mean monthly rainfall range of 20–30 mm influenced the mosquito abundance [66]. A daily rainfall range of 17–20 mm of rainfall was found to be ideal for mosquito progression and malaria spread [66].

Dommar et al. [71] modeled the chikungunya outbreak on Reunion Island between May 2005 and February 2006 using an ABM investigating the outbreak of the disease on the network with precipitation. The model revealed that the spread of chikungunya was dominated by precipitation patterns in which a quick spread of the disease was observed following the initial rainfall period, while the negligible spread was as well observed during the dry season [71].

### 3.3. Humidity

Humidity (relative or absolute) was used as an indicator in several studies and most of these studies found a positive association with arbovirus disease incidences [14,24,25,32,63,65,69,70,73,76]. Refs. [12,23,24,30,64,65,68,71] studies reviewed dengue, chikungunya, and Zika modeling using different ABM, statistical and mathematical models. Ingabire and Kimura [58] modeled malaria using Susceptible-Infected/Susceptible-Infected-Recovery model and Tourre et al. [60] modeled the climate impact on malaria in Burkina Fasso using Atlantic Multi-decadal Oscillation (AMO) and logistical regression using Arpege System 3 model.

A study from Rwanda on malaria by Ingabire and Kimura [63] showed that a decrease in humidity caused the number of malaria patients to decrease, while an increase in humidity led to an increase in the number of patients showing a correlation between humidity and infection rates of *Anopheles gambiae*. When the model was simulated using real climate data, humidity change caused the number of mosquitoes and the number of patients to change exponentially suggesting that humidity had a large impact on the mosquito increase and malaria patient increase [63]. A 60% monthly value of relative humidity provided suitable conditions for malaria diffusion [65].

Since humidity is governed by a combination of temperature and rainfall, it is potential for virus transmission because it influences the lifespan of the mosquito. Some studies have indicated that annual average water vapor pressure is a crucial climatic predictor of global dengue occurrence [73]. Humidity positively affected the number of female *Ae. Aegypti* captured until an optimal of 80% was reached [70,75,76]. In slightly urbanized sites, the relative humidity was found to positively influenced *Ae. Aegypti* immatures [24].

A study by Mulyani et al. [76] observed the influence of humidity on mosquito behavior on dengue spreading in which a humidity of greater than 70% was observed to favor reproduction, random flying, and biting behavior. Mosquitoes could only fly randomly at the humidity range of 60–70% and at less than 60% humidity the mosquitoes died. An ABM was used to demonstrate the humidity influence on temperature [76].

### 3.4. Environmental Factors

*Ae. Aegypti* immatures are more abundant in artificial and large water containers, in a study by Talaga et al. [24], the container type and size were found to influence *Ae. Aegypti* immature abundance. Water-filled tires were found to be the most favorable habitat for *Ae. Aegypti* [24]. In an experiment in Brisbane, *Ae. Aegypti* was found to survive in buckets and rainwater tanks that were exposed to the coldest water temperatures which were thought to be influenced by the high specific heat capacity and thermal mass of water. This led to the conclusion that permanent water storage containers presented ideal larval habitat for the re-establishment and persistence of *Ae. Aegypti* in Queensland, Australia [32].

Maneerat and Daudé [75] investigated the effects of various factors such as human density, breeding site density, and topology on *Ae. Aegypti* mosquito behavior using an ABM in India in which the results revealed a significant relationship between urban topology, human densities, and adult mosquito flight [75]. Model of Mosquito Aedes (MOMA) aimed to produce statistical data on mosquito behaviors and population dynamics in different geographical and climatic conditions in India. Adult mosquitoes were simulated as agents which interacted with their local environment then resources were provided for their biological development which could also constrain their flight or egg-laying behaviors [75]. The breeding sites’ behaviors were assumed to relate to temperature, precipitation, and land-use category of its spatial object [75].

The vegetation index which was an indicator of the amount of green vegetation in an area was closely related to rainfall incidences. Vegetation created suitable habitats for mosquito breeding which as a result led to an increase in mosquito density [69,72]. The growth of vegetation is in some extent regulated by temperature and rainfall. It is associated with warmer temperatures and increased rainfall, as a result an increase in vegetation indices is related to an increased number of eggs laid by mosquitoes. Vegetation cover provides suitable breeding sites which directly increases the vector abundance and the disease transmission rate [37,38,86]. Thus, vegetation index has a positive relationship to egg prediction as well as oviposition activities leading to increased vector activity due to increases in habitat for mosquitoes [38]. Normalized difference vegetation index (NDVI) and normalized difference water index (NDWI) are the two variable that have been used to determine the vegetation cover and their influence on larval survival, breeding sites, oviposition activity, vector activity, and vector population growth [34,36,38,87].

## 4. Discussion

This review has analyzed the literature on arbovirus modeling from the global perspective with emphasis on studying the techniques used in modeling the outbreaks, transmissions, and surveillance of dengue in Africa, especially SSA which modeled dengue disease. The reviewed literatures have considered climatic and environmental factors that are believed to influence the dengue disease outbreaks, transmission, and surveillance. It has come to our attention that in Africa, accurate identification of arboviral infections in resource-limited settings is difficult, and most cases are misdiagnosed as malaria [12,88]. The continued transmission of DENV on a local level, the recurring episodes of DENV as well as the wide spread of *Ae. Aegypti* and *Ae. Albopictus* in different areas of the world calls for a need of vector control and surveillance in the areas that are likely to be introduced with the DENV [10,89]. Ward and others [15] recommended that dengue should be included in the regular disease surveillance and control programs despite its scarcity of data in the African region. There is evidence on the influence of climate change on dengue epidemics and possible expansion of *Ae. Aegypti* and *Ae. Albopictus* species into the areas where the environmental conditions were not suitable for their breeding, but due to changes in climate these areas/regions are now becoming suitable for these species [16,88,90]. Since ABM is the most natural way to describe the underlying social/epidemiological system as well as a versatile and powerful platform for modeling different healthcare interventions and answering a wide range of policy making questions [5] it is crucial to utilize its benefit in the African context. Globally, disease modeling using ABM has been widely implemented, but in the African context it is still in its primary stages. Owing to the benefits of ABM, more studies need be carried out in Africa to model diseases using ABM technology. Based on the search strategies and criteria used, most of the literature obtained demonstrated the following key issues.

(i) Through the review, it has been identified that ABM in Africa is mostly implemented for other diseases such as malaria, Ebola, rift valley fever, west Nile virus, tuberculosis, human immunodeficiency virus, and other dominant diseases rather than dengue disease [62,63,64,65,66,67,68]. This shows that there is a lack of sufficient literature on ABM for dengue disease in Africa and thus points to a need for further research on dengue and ABM and simulation in the African context.
(ii) Dengue modeling in ABM is extensively conducted in non-African countries, and although extensively researched in these non-African countries, few studies have considered the influence of climatic and environmental factors. The context of non-African countries into which climatic and environmental conditions have been incorporated into dengue disease modeling is different from the African context.
(iii) Agent-based model frameworks that have been implemented in the reviewed studies included NetLogo, Repast, AnyLogic, EMOD, MESA, MASON, Mlab, Swarm, StarLogo, and Spark. Other recent and more capable ABM tools such as JADE, GAMA, WALK, MARS, and Vigueras were not found in the reviewed works of literature. Advances in technology and computing power have made emerging new tools such as MARS offer a promising output in ABM and simulation. The MARS framework developed for multi-agent research and simulations can simulate a large number of agents’ interactions using a local machine or a cloud-native environment [91,92,93]. The MARS framework allows the implementation of layered architecture and allows spatio-temporal data integration in which raster- and vector-based data can efficiently be handled. A study by Glake et al. [92] identifies that it is important to evaluate the spatio-temporal data model of the MARS framework with real-world cases.
(iv) To the authors’ knowledge, there are no available reviews that have specifically studied and modeled dengue disease and the influence of climatic and environmental factors using the MARS framework for ABM in the SSA context. Therefore, this review is crucial to enlighten a need for more studies on dengue modeling in Tanzania’s context and explore the effects of climatic, environmental, and spatio-temporal factors on dengue disease outbreaks, transmission, and surveillance.

**Figure 3:**
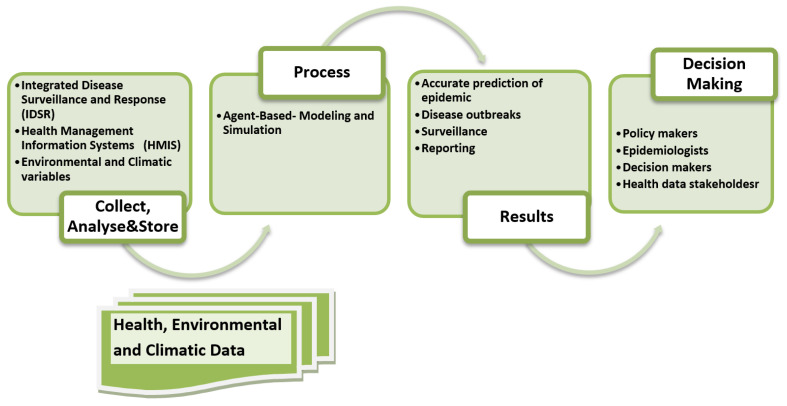
Proposed ABM model components for dengue modeling.

Integrated vector management (IVM) is a crucial component in the eradication of vector-borne diseases [94,95]. It incorporates decision making based on human and institutional resources and engage communities so as to ensure sustainability of the control strategies. Since IVM relies on an understanding of how environmental factors affect the distribution and densities of different species of vectors and how effectively control measures reduce vector–human contact, vector survival and overall intensity of pathogen transmission [94,95,96]. Owing to the numerous benefits of ABM in modeling real world systems-such as the bounded rationality, ability to capture emergent behavior, its bottom-up approach in modeling macro-systems from micro-level components, heterogeneity and discrete nature of ABMs, networked interactions among agents, completeness and flexibility of the models [47,50]. All these benefits facilitate modeling of the complicated human-vector-pathogens relationship helping to bridge the gap between what epidemiologists can deliver and what public health policy makers require [5]. Thus, it enables spatial and temporal components to be tracked, which impose challenges on surveillance of infectious diseases. Figure 3 presents a proposed ABM that incorporates necessary components that can aid in the development of descriptive and predictive models for population dynamics and transmission potential of vector-borne diseases.

## 5. Conclusions

Studies have been conducted that consider the influence of climatic factors such as temperature, precipitation, and humidity on infectious disease transmission with recent emphasis backed up with the rising concerns about climate change and the potential expansion of the geographical range of infectious diseases such as malaria, chikungunya, Zika and dengue [7,62]. In the reviewed studies, many have been demonstrated to explore the effect of temperature on arboviruses disease transmission; minimal exploration of the influence of other factors such as precipitation and humidity has been carried out. It is further observed that rainfall has been a bit more thoroughly explored in its relation to disease transmission than humidity’s influence on disease transmission. In the African context, to our knowledge, almost no works of literature have been fully identified during the review process to incorporate the climatic factors, environmental factors, and their influence on dengue transmission using ABM and simulation. This provides room for further investigations and simulations on ABM in the African context on dengue and the influence of climatic and environmental factors. As stated in a study by Anders [73] that temperature, rainfall, wind speed and humidity are indicated as crucial determinants of the geographic limits within which dengue transmission could probably be persistent [6,7,73], then it is important to consider these factors when modeling dengue in the African context using ABM.

In addition, to strengthen vector control components for vector borne diseases, it is crucial to incorporate all the necessary components of IVM so as to be able to eliminate dengue fever and other related mosquito borne diseases. Strengthening components of the vector control program such as vector surveillance, disease detection, control activities and emergence preparedness is crucial. Thus, the IVM approach will continue to be an effective and efficient strategy with great promise for disease control in Africa.

## Data Availability

Not applicable.

## Appendix A

(((Dengue[mesh] OR “Breakbone Fever” OR “Fever Breakbone” OR “Classical Dengue Fever” OR “Classical Dengue Fevers” OR “Dengue Fever Classical” OR “Break-Bone Fever” OR “Break Bone Fever” OR “Fever Break-Bone” OR “Dengue Fever” OR “Fever Dengue” OR “Classical Dengue” OR “Classical Dengues” OR “Dengue Classical” OR “Disease Virus” OR “Diseases Virus” OR “Virus Disease” OR “Virus Infections” OR “Infection Virus” OR “Infections Virus” OR “Virus Infection” OR “Viral Diseases” OR “Disease Viral” OR “Diseases Viral” OR “Viral Disease” OR “Viral Infections” OR “Infection Viral” OR “Infections Viral” OR “Viral Infection” OR “Vector Borne Disease” OR “Vector-Borne Diseases” OR “Vector-Borne Disease” OR “Vectorborne Diseases” OR “Vectorborne Disease” OR “Mosquito-Borne Diseases” OR “Mosquito-Borne Disease” OR “Mosquito Borne Diseases” OR “Mosquito Borne Disease” OR “Hemorrhagic Fever” OR “Mosquito Related Diseases” OR “ Aegypti” OR “Aedes Albopictus” OR “Dengue Virus” OR DENV) AND ((“Climatic processes” [mesh] OR “Processes Climatic” OR “Climatic Process” OR “Process Climatic” OR Temperature OR Rainfall OR Humidity OR “Relative Humidity” OR Weather OR “Spatial Epidemiology” OR “Climate Change” OR “Climatic Factors”) OR (Environment [mesh] OR Environments OR “Impacts Environmental” OR “Environmental Impacts” OR “Impact Environmental” OR “Environmental Impact” OR Vegetation OR Forest OR “Forested Areas” OR “Area Forested” OR “Areas Forested” OR “Forested Area” OR Woodland OR Woodlands OR Forestlands OR Forestland OR Plants OR Trees OR leaves OR Wetland OR “Mangrove Swamps” OR “Mangrove Swamp” OR “Swamp Mangrove” OR “Swamps Mangrove” OR “Mangrove Forests” OR “Forest Mangrove” OR “Forests Mangrove” OR “Mangrove Forest” OR Swamps OR Swamp OR Bogs OR Bog OR Marshes OR Marsh OR River OR Rivers OR “Fresh Water” OR “Burrow Pits” OR “Discarded Plastic Containers” OR “Unused Car Tires” OR “Environmental Factors”))) AND (“Agent based modeling” [mesh] OR “Analyses Systems” OR “Systems Analyses” OR “Systems Oriented Approach” OR “Approach Systems Oriented” OR “Approachs Systems Oriented” OR “Systems Oriented Approachs” OR “System Dynamics Analysis” OR “Analyses System Dynamics” OR “Analysis System Dynamics” OR “Dynamics Analyses System” OR “Dynamics Analysis System” OR “System Dynamics Analyses” OR “Analysis Systems” OR “Systems Approach” OR “Approach Systems” OR “Approachs Systems” OR “Systems Approachs” OR “Systems Thinking” OR “Systems Thinkings” OR “Thinking Systems” OR “Thinkings Systems” OR “Systems Medicine” OR “Medicine Systems” OR “Medicines Systems” OR “Systems Medicines” OR “Complexity Analysis” OR “Analyses Complexity” OR “Analysis Complexity” OR “Complexity Analyses” OR “Agent-Based Modeling” OR “Agent Based Modeling” OR “Agent-Based Modelings” OR “Modeling Agent-Based” OR “Modelings Agent-Based”)) AND (Africa[mesh] OR “Africa South of the Sahara” OR “Sub-Saharan Africa” OR “Africa Central” OR “Central Africa” OR Cameroon OR “Central African Republic” OR Chad OR Congo OR “Democratic Republic of the Congo” OR “Equatorial Guinea” OR Gabon OR “Sao Tome and Principe” OR “Africa Eastern” OR “East Africa” OR Burundi OR Djibouti OR Eritrea OR Ethiopia OR Kenya OR Rwanda OR Somalia OR “South Sudan” OR Sudan OR Tanzania OR Uganda OR “Africa Southern” OR Angola OR Botswana OR Eswatini OR Lesotho OR Malawi OR Mozambique OR Namibia OR “South Africa” OR Zambia OR Zimbabwe OR “Africa Western” OR Benin OR “Burkina Faso” OR “Cabo Verde” OR “Cote d’Ivoire” OR Gambia OR Ghana OR Guinea OR “Guinea-Bissau” OR Liberia OR Mali OR Mauritania OR Niger OR Nigeria OR Senegal OR “Sierra Leone” OR Togo).

## Appendix B

(*ALL* (Dengue *OR* “Classical Dengue Fever” *OR* “Break Bone Fever” *OR* “Dengue Fever” *OR* “Disease Virus” *OR* “Virus Disease” *OR* “Virus Infections” *OR* “Infection Virus” *OR* “Virus Infection” *OR* “Viral Diseases” *OR* “Disease Viral” *OR* “Viral Disease” *OR* “Viral Infections” *OR* “Viral Infection” *OR* “Vector Borne Disease” *OR* “Vector-Borne Diseases” *OR* “Vector-Borne Disease” *OR* “Mosquito-Borne Diseases” *OR* “Mosquito-Borne Disease” *OR* “Mosquito Borne Diseases” *OR* “Mosquito Borne Disease” *OR* “Hemorrhagic Fever” *OR* “Mosquito Related Diseases” *OR* “Aedes Aegypti” *OR* “Aedes Albopictus” *OR* “Dengue Virus” *OR* denv) *AND ALL* ((“Climatic processes” *OR* temperature *OR* rainfall *OR* humidity *OR* “Relative Humidity” *OR* weather *OR* “Spatial Epidemiology” *OR* “Climate Change” *OR* “Climatic Factors”) *OR* (environment* *OR* “Environmental Impacts” *OR* “Environmental Impact” *OR* vegetation *OR* forest* *OR* “Forested Areas” *OR* “Forested Area” *OR* woodland* *OR* forestland* *OR* plants *OR* trees *OR* wetland *OR* “Mangrove Swamps” *OR* “Mangrove Forests” *OR* “Mangrove Forest” *OR* swamp* *OR* marsh* *OR* river* *OR* “Burrow Pits” *OR* “Discarded Plastic Containers” *OR* “Unused Car Tires” *OR* “Environmental Factors”)) *AND ALL* (“Agent Based model” *OR* “Analyses Systems” *OR* “Systems Oriented Approach” *OR* “System Dynamics Analysis” *OR* “Analysis System Dynamics” *OR* “Dynamics Analysis System” *OR* “System Dynamics Analyses” *OR* “Analysis Systems” *OR* “Thinking Systems” *OR* “Complexity Analysis” *OR* “Analysis Complexity” *OR* “Complexity Analyses” *OR* “Agent-Based Modeling” *OR* “Agent Based Modeling” *OR* “Modeling Agent-Based” *OR* “Multi-Agent Systems” *OR* “Individual-Based Systems”) *AND ALL* (africa *OR* “Sub-Saharan Africa” *OR* “Central Africa” *OR* cameroon *OR* congo *OR* “Equatorial Guinea” *OR* “East Africa” *OR* eritrea *OR* ethiopia *OR* kenya *OR* rwanda *OR* sudan *OR* tanzania *OR* uganda *OR* “Africa Southern” *OR* angola *OR* botswana *OR* malawi *OR* namibia *OR* “South Africa” *OR* zimbabwe *OR* “Western Africa” *OR* benin *OR* “Burkina Faso” *OR* ghana *OR* “GuineaBissau” *OR* mali *OR* mauritania *OR* niger* *OR* senegal *OR* “Sierra Leone”.

## Appendix C

**Table A1:** Summary of reviewed articles.

Serial #	Author, Article Type, Diseases Studied	Climatic Factor Temperature, Rainfall/Precipitation, Humidity/Relative Humidity)	Environmental Factors	The Modeling Approach Used (Agent-Based, Compartmental Models, Machine Learning, Time Series, etc.)	Research Settings (Africa, Global, Country-Specific, etc.)	Study Objective and Results
1.	Wearing et al. [69], Review paper, Dengue and chikungunya.	Effects of humidity, temperature, and rainfall on egg diapause and adult survival.	Vector density/ population dynamics, urbanization, availability of man-made containers, vegetation index.	Review of different models: process-based models, statistical models, mathematical models.	Global	The study observed that when a suitable mosquito vector (*Ae. Albopictus/Aegypti*) coupled with suitable conditions for their survival and transmission was required for the occurrence of a mosquito-borne virus in a population. The study also revealed that the virus was introduced from external sources and conditions amenable to its transmission.
2.	Talaga et al. [24], Experimental. Paper, *Ae. Aegypti*.	Daily precipitation, air temperature, wind, and relative humidity.	Artificial water containers (such as CDC ovitraps, and car tires), natural water containers (such as native tank bromeliads, dry stamps of bamboo), size of aquatic habitat, and an abundance of *Ae. Aegypti* immatures.	Multi-model inference approach.	French Guiana (Kourou which is a small Neotropical), October 2013–October 2014.	The relative influence of biotic and abiotic parameters on the immature population of *Ae. Aegypti* was explored using four different types of water containers and three urbanized sites. The study found that artificial water containers, size of aquatic habitat, amount of precipitation, temperature, and relative humidity positively influenced the number of *Ae. Aegypti* immatures. The study presented co-existence of *Ae. Aegypti* with predators and competitors on the abundance of immatures.
3.	Center [72], Annual Meeting Report, Female and male *Ae. Aegypti*, Malaria parasites.	Temperature (low 19 °C and high 30 °C).	288 Vegetation zones, 93 urban zones, breeding spots, 50 households.	ABM	Key West, Florida with an area of approximately 30,000 m^2^ with around 50 households.	An ABM coupled with a geographic information system for identifying zones with resources necessary for the survival of both male and female *Ae. Aegypti* and modeling possible breeding site locations. Results showed that the spatial distribution of the vegetation zones, urban zones, and breeding spots together with the temperature, constrained the population of *Ae. Aegypti* to a mean high of around 2000 during the fall season and around 600 in the late winter season.
		Tropical climate, weather.	Demographics data.	ABM (EMOD framework).	SSA	EMOD is a proprietary epidemiological modeling software package that is used to determine the best combination of interventions that will eventually eradicate the disease. EMOD is a human, mosquito, and parasite modeling program that takes mosquito, weather, demographic, and parasite parameters as input.
4.	Ingabire and Kimura [63], Journal Article, Malaria (*Anopheles gambaie*).	Temperature (minimum 10 °C and maximum 29 °C), humidity (30–97%), rainfall.	N/A	Mathematical SIR/IR model.	Rwanda meteorology data for 2017 was used.	The research focused on climate effects on the sensitivity of a reproduction rate, death rate, and infection rate, focusing on the life cycle of mosquitoes based on the change in temperature and humidity. Mosquito reproduction increased when humidity was more than 80%. Thus, the high birth rate during hot and high humidity season could accelerate the increase in mosquitoes and enhance the spread of infection. Simulation with real data showed that the number of mosquitoes and the number of patients changed exponentially was mainly caused by humidity change.
5.	Anders [73,83,84], Ph.D. Thesis, Journal articles, Dengue.	Temperature, rainfall, humidity.	Breeding sites, mosquito density, human population density, water storage needs, and practices.	Mathematical modeling.	Clinically diagnosed dengue cases that were admitted between 1996 and 2009 in three hospitals in Ho Chi Minh City, Southern Vietnam.	The study sought to characterizethe distribution of dengue fever as well as the factors that influence individual and population-level infection risk and disease outcome. Heterogeneity in dengue incidence in space and time is studied. Dengue is found to be sensitive to climatic conditions, which influence virus replication, development, and vector survival. Non-climatic variables such as environmental changes, population growth and human movement, work hand in hand with the climatic variables.
6.	Liu [25,97,98], Ph.D. Thesis, Journal articles Dengue, Zika.	Temperature (daily, min, max, average), precipitation, relative humidity.	N/A	Multivariate Exponentially Weighted Moving Average (MEWMA) model with a forward feature selection (FFS) algorithm (MEWMA-FFS) framework, machine learning.	Detection of large dengue outbreaks in San Juan-Puerto Rico 2004–2017, Iquitos-Peru 2004–2013, Mexico country 2004–2013.	The study focused on two aspects of emerging and re-emerging infectious disease surveillance systems, with the goal of developing a method for detecting emerging and re-emerging outbreaks as early and accurately as possible, and the study has assessed the tradeoff between the complexity of the model and prediction reliability. The results showed that in Mexico, a subset of the climate-relevance time series model was the best option for early detection of large dengue outbreaks, as was the case in San Juan, where the climate system’s performance was more robust across cross-validation and out-of-sample testing periods. In Iquitos Peru, due to its location, experienced a tropical rainforest climate that lacks a distinct dry season demonstrating that climate was not a good predictor for large dengue outbreaks.
7.	Deza-Cruz [70], Ph.D. Thesis, Dengue, chikungunya, Zika.	Humidity, temperature, rainfall, wind speed.	Household (water supply, air-conditioning, intact mosquito screens, construction materials, number of residents in the house, number of rooms in the house), mosquito larval habitat (Trash collection, Pools water, Storage water, Debris around house, Plant pots).	Machine learning.	University students at St. Kitts and Nevis between September 2014–May and 2015.	The study found that arbovirus transmission was associated with climatic variables connected to seasonality, which are temperature and rainfall. Humidity and temperature had a positive influence on disease transmission, rainfall had a dual effect (much rain caused a flushing effect, and moderate rain had a positive effect). At the mean temperature of 26 °C and 29 °C large number of mosquitoes were captured, while the number declined at 31 °C.
8.	Trewin [32,33,99], Ph.D. Thesis, Journal Articles, *Ae. Aegypti*.	Temperature, rainfall, the humidity of the water in the rainwater tanks and the buckets.	Rainwater tanks, buckets.	ABM (Repast Simphony 2.4.0)	Datasets from Brisbane city council 2012, Queensland government 2017 and Australian Bureau of Statistics 2011.	The model aimed at understanding the implications of mosquito spread between rainwater tanks and identifying risk areas in Brisbane. The number of rainwater tanks in the landscape was principally responsible for the risk.
9.	Tourre et al. [65], Journal article, Malaria.	Temperature (minimum, maximum), rainfall, relative humidity.	N/A	Impact model to evaluate the influence of climate conditions on malaria risk.	Nouna region in northern Burkina Faso. Rainfall, temperature, and relative humidity data for the period of 1983–2011 were used.	Climate impact model for malaria risk. Total rainfall for three months was found to be a confounding factor for the malaria vector density. It was discovered that there is a substantial relationship between risk for malaria and low-frequency rainfall variability related to the Atlantic Multi-decadal Oscillation (AMO).
10.	Borges et al. [74], Conference paper, Dengue (*Ae. Aegypti* pupal productivity).	N/A	Type of container.	ABM using NetLogo.	The model is simulated 1500 times each simulated 100 days.	ABM for evaluating the Pupal Productivity of *Ae. Aegypti* in containers. The model took into account the pupae production capability of each container in which the mosquitoes laid eggs. The model results were compared to the simulation’s average percentage of pupae per container and the percentage of container productivity defined in Brito-Arduino 2014.
11.	Mukhtar et al. [66], Journal Article, Malaria (*Anopheles gambiae*).	Temperature, rainfall.	N/A	Mathematical models.	Two regions in South Sudan.	Climate-driven dynamics model was used to evaluate the impact of rainfall and temperature on the dynamics of the mosquito population in a specific region of South Sudan. Daily temperatures ranged 20 °C to 35°C and rainfall ranged 17 mm to 20 mm were ideal for mosquito progression which facilitated malaria spread. Thus, the warmer temperature might lead to disease intensification.
12.	Rumisha et al. [67], Journal Article, Malaria (*Anopheles funestus*, *Anopheles gambiae*, *Anopheles arabiensis*).	Rainfall, temperature.	N/A	Bayesian spatio-temporal binomial and zero-inflated negative binomial regression models, Machine learning model.	Data are collected for three years by Demographic Surveillance System (DSS) in Rufiji Tanzania.	The study aimed to assess spatial-temporal variation and heterogeneity of malaria transmission in the Rufiji DSS site using a large geo-referenced biweekly entomological dataset collected over three years (October 2001–September 2004) and rigorous Bayesian geostatistical models. Results depicted temporal and seasonal variation in entomological inoculation rate along the study period and study area.
13.	Kang and Aldstadt [26], Journal Article, Dengue.	N/A	Mosquito habitats, the spatial configuration of buildings (model environment had 4 schools, 20 workplaces and 895 houses).	ABM using AnyLogic.	A portion of Kamphaeng Phet Province (KPP), Thailand. Parts of a registered residents dataset of KPP in 2009 were used.	The study sought to shed light on the significance of specifying building spatial configurations and mosquito habitats. The experiments’ findings revealed a significant influence on human residential and mosquito population patterns.
14.	Maneerat and Daudé [75], Journal Article, Dengue (*Ae. Aegypti* female mosquito).	Daily time, hourly temperature and daily rainfall.	Urban landscape.	ABM-Model of Mosquito Aedes (MOMA).	Hauz Rani is a neighborhood in south Delhi (India). Meteorological data of June 2008 is used for performing the simulations.	A behavioral model MOMA of the *Ae. Aegypti* mosquito aimed to study the effects of factors such as breeding site density, human density, and topology at a neighborhood level on the dynamics of mosquito population. MOMA investigated mosquito population dynamics in a variety of urban settings. The findings revealed a link between human density, urban topology, and adult mosquito flight.
15.	Dommar et al. [71], Journal Article, Chikungunya.	Precipitation.	N/A	ABM	An agent-based model, 2 years (28 March 2005–12 February 2006) daily precipitation data for La Réunion.	The researchers created an ABM to investigate the spatiotemporal heterogeneity of an infectious vector-borne disease outbreak. The impact of precipitation-dependent vector populations and the structure of the underlying network topology on epidemiological dynamics was investigated using a model. Results indicated that precipitation was the dominant factor influencing the spatio-temporal transmission.
16.	Mulyani et al. [76], Journal Article, Dengue.	Temperature, humidity.	N/A	ABM-NetLogo.	Data and information from the national meteorology, climatology and geophysics agency were used. Community health data of Bogor, a region in Dramaga for two periods, January–June 2015 and July–December 2015 were used.	The paper has focused on the simulation behavior of the agent, agent interactions, and agent-environment interactions. Temperature and humidity variables have been used. Results showed that with optimal parameters of temperature and humidity the percentage of infected humans increased as well as mosquito count increased. With low parameters, the percentage of infected humans was steady or did not happen, while the number of mosquitoes decreased. Meanwhile, with fluctuated parameter values, the infection of humans and mosquitoes also fluctuated.
17.	Deng et al. [77], Conference Paper, Dengue.	Wind direction	Landscape roughness, population, and land use type.	ABM developed with VB6.0	Virtual environment.	The spreading mechanism of dengue is explained through agent-agent and agent-environment interactions.
18.	Mahmood et al. [14], Book Chapter, Dengue.	Temperature, rainfall, humidity.	Water bodies, Mosquito density.	ABM using AnyLogic university edition.	Dengue outbreak data from Islamabad city in Pakistan, 2013.	Results showed that dengue transmission was dependent on temperature, when temperature increased, the biting rate of *Ae. Aegypti* increased which resulted in increased dengue cases.
19.	Rodríguez [78], Ph.D. Thesis, Dengue.	Temperature, precipitation.	Socio-economic, demographic variables.	Dengue Fever ABM (DFABM) was developed using the Java programming language and open-source MASON simulator, a multi-threaded agent-based simulation platform.	Datasets on monthly reported dengue cases, weather predictors (temperature and precipitation), socio-economic and demographics measures of the population of Central Valley of Costa Rica from 1993 to 2008.	The spread of dengue fever in ABM was simulated using GIS in an urban setting while also taking into account individual interactions in a geospatial context. The variables of temperature, precipitation, socioeconomic status, and demographics were examined. The results showed that high temperatures with heavy precipitation affected the greatest proportion of reported dengue cases. In general, high temperatures, poor housing conditions, and male predominance during warm seasonal rainy periods created ideal conditions for mosquito outbreaks and, as a result, occurrences and rates of dengue cases.
20.	Colón-González et al. [68], Journal Article, Malaria.	Air temperature, rainfall.	Urbanization, rural districts, socioeconomic indicators.	Time series cross-validation algorithm.	Malaria incidences in Rwanda and Uganda from 2002 to 2011.	The study looked at the short-term effects of rainfall, air temperature, and socioeconomic indicators on malaria incidence. It emphasized the importance of using climatic information in the analysis of malaria surveillance data and demonstrated the potential for the development of climate-informed malaria early warning systems.
